# Consumption of edible oil blended with flax, coconut, sunflower, and olive oil can significantly improve the negative health consequences of high-fat/high-cholesterol diet in Sprague Dawley rats

**DOI:** 10.3389/fnut.2024.1469601

**Published:** 2024-09-20

**Authors:** Roshina Rabail, Ammar B. Altemimi, Cristina Maria Maerescu, Claudia Terezia Socol, Florin Leontin Criste, Abdur Rauf Khalid, Mahwish, Shahzad Hussain, Zhi-Wei Liu, Rana Muhammad Aadil

**Affiliations:** ^1^National Institute of Food Science and Technology, University of Agriculture, Faisalabad, Pakistan; ^2^Department of Food Science, College of Agriculture, University of Basrah, Basrah, Iraq; ^3^College of Medicine, University of Warith Al-Anbiyaa, Karbala, Iraq; ^4^Department of Genetics, Department of Animal Science and Technology, University of Oradea, Oradea, Romania; ^5^Department of Livestock and Poultry Production, Faculty of Veterinary Sciences, Bahauddin Zakariya University, Multan, Pakistan; ^6^Institute of Food Science and Nutrition, University of Sargodha, Sargodha, Pakistan; ^7^Department of Food Science and Nutrition, College of Food and Agriculture, King Saud University, Riyadh, Saudi Arabia; ^8^College of Food Science and Technology, Hunan Agricultural University, Changsha, China

**Keywords:** blended cooking oil, anti-oxidation, anti-inflammatory, hyperlipidemia, health blended cooking oil, high-fat-high-cholesterol diet, cardiovascular risk indices, echocardiography

## Abstract

**Background:**

Increasing cardiac, hepatic, and metabolic diseases have raised the need to modify our contemporary lifestyles toward balancing and diversifying the nutrients in our daily diet. Objective: Dietary fats should be modified to healthier versions by blending different vegetable oils. Therefore, in this study, an oil blend with health-protective and promoting fatty acid combinations was investigated to bring down the progression of cardiac and other metabolic diseases.

**Methodology:**

A bio-efficacy trial was performed to investigate the therapeutic potential of an oil blend in 30 hyperlipidemic rats. Five rats were allocated to each group (coconut, flaxseed, olive, sunflower, and blended oil) for 42 days and were compared with the initial values of hyperlipidemic rats. Methodological investigations were performed for the body weight, naso-anal length, various obesity indices, visceral fat accumulation, blood and serum, cardiovascular risk indices, and echocardiograph.

**Results:**

Blended oil consumption indicated significant reductions of 53.12% in body fat content (3.98 ± 0.96), 6.82% in Lee index (289.60 ± 8.27), 16.84% in BMI (0.15 ± 0.003), 57.37% in total cholesterol (52.00 ± 9.03), 68.57% in triacylglycerides (99.00 ± 9.19), 61.16% in atherogenic index (0.88 ± 0.12), and 58.72% in coronary risk index (2.88 ± 0.12), when compared with the initial values.

**Conclusion:**

Blended oil consumption has significantly reduced various obesity indices, improved lipid profile, and provided significant protection against cardiovascular risk indices. Moreover, the results of blended oil indicated significant health protective ameliorations in electrocardiographs. Its regular consumption could help to reduce the onset of obesity and metabolic diseases.

## Graphical abstract

Current dietary fats should be modified to healthier versions by blending different edible oils. A blended edible oil made from coconut oil, flaxseed oil, olive oil, and sunflower oil has shown significant improved cardiac, hepatic, renal and anthropometric biomarkers as indicated in graphical abstract.

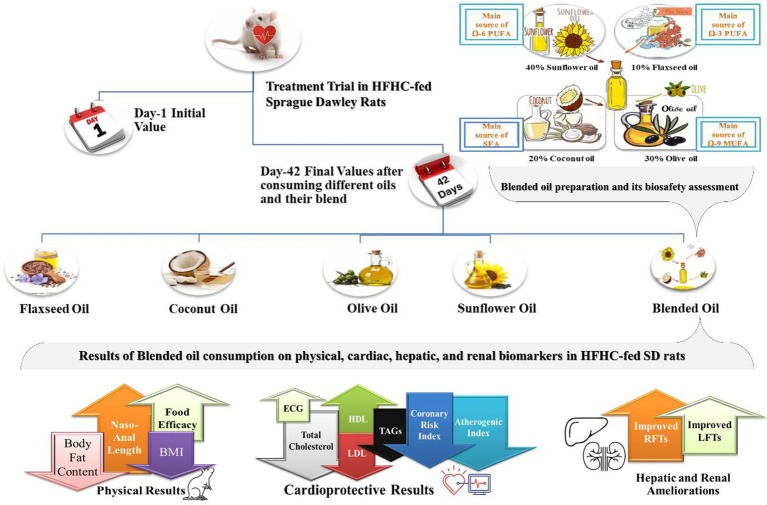

## 1 Introduction

Cardiovascular diseases (CVDs) are at the top of the list and are a serious public health concern worldwide. In 2016, CVDs were responsible for the deaths of 17.9 million people, while this rate has been raised to over half a billion people worldwide, accounting for 20.5 million deaths in 2021—nearly a third of all fatalities—and contributing to an overall rise in the predicted 121 million deaths from CVDs (1, 2). Hepatic disorders, together with CVDs, are among the primary causes of morbidity and mortality worldwide. Moreover, the rising burden of diseases has highlighted the field of nutrition and has directed food experts to bring forth natural preventative options (3). Because of the high risks of fatalities and disease progression, health practitioners must identify at-risk patients and underline the importance of crucial change initiatives in healthy eating and lifestyle (4). Lifestyle and food choices have been linked as important contributors to these CVDs, as the growing consumption of inappropriate dietary fats with imbalanced fatty acid (FA) composition in modern society has been identified as the major cause of these diseases (5). Such dietary consumption of unhealthy fats has also been linked to an onset of other metabolic diseases as well, including non-alcoholic fatty liver disease, hypertension, and obesity (6). To avoid these health problems, it is therefore more feasible to adopt a healthy diet rich in vegetable oils (3). The reduced consumption of saturated FA (SFAs) and trans fats, as well as low to moderate intake of simple carbohydrates and animal proteins, is encouraged, while increased consumption of plant-based proteins, dietary fiber, monounsaturated FA (MUFAs), and polyunsaturated FA (PUFAs), especially omega-3 (*Ω*-3) PUFA is advised (7). Such dietary advances are only conceivable if vegetable oils with healthy and balanced FA profiles are consumed (8).

According to the most recent dietary recommendations of the World Health Organization (WHO) and the American Heart Association (AHA), the proportions of SFA, MUFA, and PUFA in dietary fats should be 1:1.5:1 for individuals to maintain optimal health status (9). As a result, it is advised to eat reduced SFAs and trans fats, while increasing consumption of good dietary fats such as MUFAs, PUFAs, and considerable amounts of *Ω*-3 PUFA (7). Therefore, such dietary improvements are attainable only by ingesting vegetable oils with healthy and balanced FA profiles (8, 10–13). Making a physical blend of several naturally occurring edible vegetable oils in the appropriate ratios is a simple and alternative technique for obtaining such a healthy oil composition (14–18). This revolutionary procedure, known as oil blending, can potentially boost the nutrient content and antioxidant capacity of edible oils (19, 20).

To provide consumers with diversification that is both health-promoting and protective, innovative nutritious formulations of edible oil blends should replace the contemporary edible fats and oil trends in our food sector, especially the oil industry. Such blended edible oils can bring forward health-protective and promotive FA combinations. An oil blend was developed in our previous study using 10% flaxseed, 20% coconut, 30% sunflower, and 40% olive oils, which revealed enhanced FA composition and FA nutritional indices of (SFA:MUFA:PUFA = 1:2:1), (MUFA/PUFA = 1:2.7), and (*Ω*-3/Ω-6 ≈ 2), which were quite close to the recommendations of WHO and AHA (21). The development and marketing of an edible oil blend with FA profile and FA nutritional indices of SFA:MUFA:PUFA, Ω-3:Ω-6 PUFA, and medium chain FAs (MCFAs) that are very close to those suggested by WHO and AHA can be a game changer in the prevention and treatment of cardiovascular diseases and associated metabolic health issues. Based on this hypothesis, efficacy studies were conducted in which the novel oil blend (21) was investigated for its bio-safety in healthy rats, and its bio-efficacy in therapeutic evaluation in high-fat/high-cholesterol (HFHC)-fed hyperlipidemic rats. This part includes the study conducted on HFHC-fed hyperlipidemic rats.

## 2 Materials and methods

### 2.1 Procurement of raw materials, development of oil blend, and thermal treatment

In July 2022, high-quality sunflower (Orisun-701) and flaxseeds (Chandni) were obtained from the Ayub Agricultural Research Institute in Faisalabad, Pakistan, and dried coconut (Khoppa) was obtained from a local market in Faisalabad, Pakistan. Cold extraction by low-resistance expeller pressing machines available in the Faisalabad markets was used to collect oil from each of the three sources. The freshly cold extracted oil from olives (Bari Zaitoon 2) was provided by the Barani Agricultural Research Institute in Chakwal, Pakistan. Until further research, all four oil types were stored in inert stainless steel canisters in the dark, at room temperature. The oil blend was formed by combining 10% flaxseed oil, 20% coconut oil, 30% sunflower oil, and 40% olive oil for approximately an hour at a steady temperature of 40°C, as reported by Grover et al. (15). All the oils were heated for 30 min on an electric induction hot plate, maintaining the temperature at 120–150°C, as previously done by Kumar et al. (22).

### 2.2 Therapeutic assessment in high-fat/high-cholesterol fed rats

A 42-day efficacy study on 30 HFHC-fed female Sprague Dawley (SD) rats named the “treatment trial” was carried out to examine the potential benefits of blended oil for CVD mitigation compared to the intake of individual vegetable oils in the diet. This treatment trial was intended to evaluate the basic hypolipidemic, anti-obesogenic, and hepatoprotective effects of oil blends against various single edible oils. Rats were kept in the animal room at the University of Agriculture, Faisalabad, under the National Institute of Food Science and Technology, with the consent of the Institutional Biosafety and Bioethics Committee (D#6894/ORIC) University of Agriculture, Faisalabad, Pakistan, following National Institutes of Health guide for the care and use of laboratory animals (NIH Publications No. 8023, revised 1978). Metallic wire cages of one square foot in height and width were used with enough ventilation, 25–30°C, and a 12-h light-and-dark cycle. Water and food were freely available (23). One group of five rats was suitably kept in a single labeled cage. Prior to the start of the trial, rats were acclimatized for 2 weeks by giving them the standard basic chow diet. After that, they were subjected to the hyperlipidemia induction period by feeding an HFHC diet for a period of 21 days (contents for BCD and HFHC diet are tabulated in Table 1).

**Table 1:** Ingredients and nutritional contents of basic chow diet and high-fat/high-cholesterol (HFHC) diet.

Basic chow diet		Formulation of HFHC diet from basic chow diet			
Ingredients	(g/Kg)	Contents	(g/Kg)	(%)	(kcal)
Maize	620	Basic chow diet	700	70	203
Soya bean meal	180	Cholesterol	20	2	18
Wheat offal	40	Banaspati ghee	280	28	252
Full-fat soya	130.5				
Bone meal	20.5				
Lysine	2				
Methionine	3				
Salt	4				
Total	1,000	Total	1,000	100	473
Nutritional contents in basic chow diet			Nutritional contents in HFHC diet		
Contents	(g or Kcal / Kg)	(%)	(g or Kcal/Kg)	(%)	
Moisture (g/kg)	120	12	80	8	
Protein (g/kg)	220	22	150	15	
Fat (g/kg)	20	2	450	45	
Cholesterol (g/kg)	0	0	20	2	
Fiber (g/kg)	60	6	40	4	
Ash (g/kg)	80	8	5.5	5.5	
Energy kcal/kg	2,900		4,730		
Energy kcal/g	2.9		4.73		
Energy kcal/100 g	29	29	47.3	47.3	

### 2.3 Experimental design and preparation of diets

After the completion of the hyperlipidemia induction period, one group G0 of five rats was decapitated to get initial (day 1) samples and vital organs for comparing the improvements in hyperlipidemic biomarkers achieved by different oil groups at the end of the study. The remaining rats were weighed and randomly assigned to five oil groups (5 **×** 5 = 25). These groups were given 100% oil replacement in a basic diet, where G1 was given coconut oil, G2 was given flaxseed oil, G3 was given olive oil, G4 was given sunflower oil, and G5 was given blended oil. Experimental diets of rats with minor alterations and oil replacements are provided in Table 2.

**Table 2:** Experimental diets for treatment trial rats.

Ingredients	Groups				
	G1	G2	G3	G4	G5
	(g/Kg)	(g/Kg)	(g/Kg)	(g/Kg)	(g/Kg)
Corn starch	650	650	650	650	650
Casein	80	80	80	80	80
Flaxseed oil	120	–	–	–	–
Coconut oil	–	120	–	–	–
Sunflower oil	–	–	120	–	–
Olive oil	–	–	–	120	–
Blended oil	–	–	–	–	120
Wheat bran	130	130	130	130	130
Vitamin min. mix.	20	20	20	20	20
Total amount	1,000	1,000	1,000	1,000	1,000
Energy kcal/100 g	400	400	400	400	400
Energy kcal/g	4	4	4	4	4
Energy kcal/kg	4,000	4,000	4,000	4,000	4,000

### 2.4 Clinical assessment

#### 2.4.1 Assessment of physical indicators

Food consumption was recorded daily, and body weight was measured at weekly intervals. Fat intake, food efficacy ratio (FER), body fat content, Rõhrer, TM, Lee, and Body Mass Indexes were computed at the end of the trial (24–27). After the experiment concluded, the rats were made unconscious by placing them by placing them in an anesthesia-producing chamber by administering chloroform. The hearts of rats were punctured to obtain a blood sample as soon as they appeared to be unconscious. The rats were killed and their vital organs (heart, liver, kidneys, spleen, visceral fats, and small and large intestines) were removed after their blood was drawn. The organ weight was calculated as g/100 g. Visceral fat deposition was measured by weighing visceral fat tissues carefully removed from the visceral cavity.

FER (%) = [(Body weight gain/day ÷ Food intake/day) × 100].

Fat Intake (%) = [(12% × Accumulated food intake × 1,000) ÷ (Final body weight × Days)].

Lee index = [Body weight (g)^1/3^ ÷ Naso-anal length (cm)] × 10^3^.

BMI = [Body weight (g) ÷ length (cm^2^)].

Body fat content = 0.581 × TM index - 22.03.

Rõhrer index = [Body weight (g) ÷ Naso-anal length (cm)^3^] × 10^3^.

TM index = Body weight (g) ÷ Naso-anal length (cm)^2.823^ × 10^3^.

#### 2.4.2 Assessment of biochemical indicators

Blood was drawn via cardiac puncture at the start and end of the experiment to perform the required biochemical assays. Using commercial kits from Merck, Germany, blood hematology, blood glucose, lipid profile, liver function tests (LFTs), and renal function tests (RFTs) were performed in compliance with Ogunlana (28) and Ismail (29) procedures. For the hematological assay, blood samples were obtained in tubes containing EDTA (30). Using an automatic hematological analyzer, as previously done, the effects of oil consumption on red blood cells (RBC), hemoglobin (Hb), hematocrit concentration (HCT), total leukocyte count (TLC), mean corpuscular volume (MCV), mean corpuscular hemoglobin (MCH), and platelets were examined (31, 32).

Blood samples were taken for serum-based analysis in yellow vacuum collection tubes that were used for serum separation and were not heparinized. The blood was then allowed to clot at room temperature for 15–30 min before the serum was extracted into an Eppendorf tube using a 10-min centrifugation at 4°C and 2000 rpm. The serum was carefully separated and used to measure LFTs, RFTs, lipid profile, and blood glucose. Using a commercial kit from Merck, the lipid profile was measured calorimetrically for total cholesterol (TC), triacylglycerides (TAGs), and high-density lipoprotein (HDL) cholesterol (33). The remaining two lipid profile components, however, were calculated using the formulas (LDL = TC–HDL–VLDL) and (VLDL = TAGs/5), respectively, to determine the levels of low-density lipoprotein (LDL) and very-low-density lipoprotein (VLDL) cholesterol (mg/dl) (27, 34). While using Merck’s commercial diagnostic kit based on the calorimetric approach, the LFTs, including alanine transaminase (ALT) and aspartate transaminase (AST), and RFTs, including total protein, albumin, blood urea nitrogen, and creatinine, were also estimated following the protocols of Ogunlana (28); Mohamed et al. (34).

#### 2.4.3 Assessment of cardiovascular risk indices

The coronary risk index (CoRI), atherogenic index (AI), and cardiovascular risk index (CaRI) were calculated using the formulas listed below (35).

Atherogenic index (AI) = LDL ÷ HDL.

Coronary risk index (CoRI) = TC ÷ HDL.

Cardiovascular risk index (CaRI) = TAGs ÷ HDL.

#### 2.4.4 Echocardiography analysis

An echocardiogram (ECG) analysis was performed on day 30 of the trial to look for any further effects of oil consumption on heart function. Rats were exposed to chloroform within a chamber that produced anesthesia for a certain amount of time. The rats were placed in a dorsal recumbent position and fastened to the operating table for the ECG procedure as soon as they displayed the first symptoms of unconsciousness. The right hind leg and forelegs were fitted with electrodes from an ECG machine PL26T04 (LTS). ECG signals were recorded using a PowerLab data acquisition device (ML856) by Animal Bio Amp. AD Equipment (MLA-136) and a digitizer (PowerLab 26 T, AD Instrument). Using LabTutor software, which offered automated data collection on the heart rate and number of normal complexes, the digitized ECG was assessed. The heart rate (HR) is the total number of cardiac contractions during a predefined period, often 1 min. The RR interval is the space between consecutive R wave peaks. Under physiological conditions, the following formula can be used to calculate HR from RR interval: HR = 1,500/number of RR interval small boxes, or HR = 300/number of RR interval small boxes multiplied by 0.2, or HR = 60/(RR interval in seconds), and it can also be computed using the Omni online ECG HR calculator (36). The HR in beats per minute (bpm), the length of the QRS complex, and the RR interval in milliseconds (ms) were reported (37).

### 2.5 Statistical analysis

All of the analyses were performed in triplicates to get their mean values ± SD. Statistical design for Bartlett’s test to test the homogeneity of variances, one-way ANOVA, Latin square design (LSD) to check difference among various treatments, and Duncan’s multiple range test to compare means were applied at 95% (*p* ≤ 0.05) confidence interval level were applied using IBM^®^ SPSS^®^ Modeler 16.0. The percentage increase or decrease (Effect) was calculated using the Omni online percentage increase calculator by using the given formula. Results were evaluated in triplicates to determine mean values and standard deviations (5, 38).

Effect = [(day 42 - day 1) × 100] ÷ day 1.

## 3 Results

### 3.1 Physical indicators

The results for physical indicators noted during the treatment trial are presented in Table 3. Results for body weight gain indicated a minimum weight gain of 80.40 ± 24.96 g per trial or 1.92 ± 0.59 g per day for coconut oil consumption, followed by 102.00 ± 24.68 g per trial or 2.43 ± 0.59 g per day for flaxseed oil consumption. A maximum weight gain of 117.00 ± 15.49 g per trial and 2.78 ± 0.36 g per day was reported for sunflower oil consumption. While blend group indicated an increase of 110.40 ± 10.83 g per trial and 2.62 ± 0.26 g per day.

**Table 3:** Effect of study oils and their blended oil consumption on physical biomarkers in high-fat/high-cholesterol diet rats.

	G0	G1	G2	G3	G4	G5	*F* value	G1 CO%↑	G2 FO%↑	G3 OO%↑	G4 SO%↑	G5 BO%↑
BW 1 (g)	127.00 ± 11.48^a^	107.40 ± 4.34^b^	120.80 ± 5.63^ca^	108.00 ± 5.15^db^	109.00 ± 6.70^eb^	100.40 ± 10.04^fb^	8.17^***^					
BW 2 (g)	166.20 ± 7.46a	187.80 ± 21.15^ba^	222.80 ± 21.47^c^	215.80 ± 18.59^dc^	226.00 ± 19.81^ec^	210.80 ± 9.14^fc^	9.09^***^	12.99↑	34.05↑	29.84↑	35.98↑	26.84↑
BW gain/trial (g)	37.20 ± 4.76^a^	80.40 ± 24.96^b^	102.00 ± 24.68^cb^	107.81 ± 17.69^dc^	117.00 ± 15.49^ec^	110.40 ± 10.83^fc^	13.86^***^	116↑	174↑	189.7↑	214.4↑	196.7↑
BW gain/day (g)	0.87 ± 0.12^a^	1.92 ± 0.59^b^	2.43 ± 0.59^cb^	2.57 ± 0.42^dc^	2.78 ± 0.36^ec^	2.62 ± 0.26^c^	13.96^***^	120.06↑	178.32↑	194.15↑	219.26↑	201.37↑
BW gain (%)	39.00 ± 16.05^a^	75.35 ± 27.09^b^	85.05 ± 23.19^cb^	101.95 ± 17.22^dc^	107.30 ± 11.81^ec^	111.46 ± 21.21^fde^	9.13^***^	93.17↑	118.07↑	161.41↑	175.12↑	185.79↑
Initial food intake/day (g)	23.59 ± 2.31^a^	27.13 ± 1.38^b^	26.26 ± 2.62^cab^	27.93 ± 0.98^db^	25.13 ± 3.72^eab^	27.26 ± 0.95^fb^	2.60^*^	15↑	11.32↑	18.4↑	6.53↑	15.56↑
Final food intake/day (g)	25.03 ± 0.78^a^	27.39 ± 4.05ba	28.21 ± 2.01^cb^	29.72 ± 2.01^db^	28.06 ± 0.76^eb^	30.06 ± 2.07^fb^	3.29^*^	9.42↑	12.7↑	18.73↑	12.11↑	20.09↑
FER (%)	3.53 ± 0.62^a^	7.31 ± 3.33^b^	8.64 ± 1.83^cb^	8.69 ± 1.83^db^	9.93 ± 1.45^ecdf^	8.77 ± 1.11^fb^	6.52^**^	107↑	144.8↑	146.2↑	181.3↑	148.4↑
Accumulated food Intake (g)	1051.50 ± 32.76^a^	1,150 ± 169.89^ba^	1185.1 ± 84.21^cb^	1248.6 ± 84.28^db^	1178.7 ± 31.94^eb^	1262.7 ± 87.12^fb^	3.29^*^	9.36↑	12.7↑	18.74↑	12.09↑	20.08↑
Fat intake (%)	18.11 ± 0.89^a^	17.79 ± 3.78^ba^	15.94 ± 1.10^ca^	16.64 ± 1.89^da^	15.00 ± 1.47^ecdf^	17.43 ± 1.34^fa^	1.77^NS^	1.77↓	12.53↓	8.12↓	17.17↓	3.75↓
Naso-anal length (cm)	17.40 ± 0.41^a^	19.30 ± 0.27^b^	20.00 ± 0.94^cb^	20.10 ± 0.41^dc^	20.20 ± 0.75^ecf^	20.00 ± 0.35^fbd^	17.40^***^	10.92↑	14.94↑	15.52↑	16.1↑	14.94↑
Full length (cm)	29.50 ± 1.00^a^	34.40 ± 0.65^b^	37.10 ± 2.43^c^	37.00 ± 0.35^dc^	35.00 ± 2.89^ebc^	36.40 ± 0.55^fbc^	15.20^***^	16.6↑	25.76↑	25.4↑	18.64↑	23.4↑
Lee index	310.8 ± 11.50a	291.29 ± 10.86^b^	297.67 ± 7.03^cb^	293.08 ± 11.78^db^	296.09 ± 7.51^eb^	289.60 ± 8.27^fb^	3.11^*^	6.28↓	4.23↓	5.7↓	4.73↓	6.82↓
Rõhrer index	31.71 ± 3.46^a^	26.13 ± 2.88^b^	27.87 ± 1.95^cb^	26.66 ± 3.22^db^	27.44 ± 2.11^eb^	26.35 ± 1.02^fb^	3.22^*^	17.6↓	12.1↓	15.93↓	13.47↓	16.9↓
TM index	52.53 ± 5.54^a^	44.12 ± 4.86^b^	47.35 ± 3.02^cab^	45.34 ± 5.34^db^	46.70 ± 3.42^eb^	44.78 ± 1.66^fb^	2.63^*^	16↓	9.89↓	13.7↓	11.1↓	14.75↓
Body fat content	8.49 ± 3.21^a^	3.53 ± 2.80^b^	5.43 ± 1.72^cab^	4.31 ± 3.10^db^	5.10 ± 1.98^eb^	3.98 ± 0.96^fb^	2.67^*^	58.4↓	36.04↓	49.23↓	39.92↓	53.12↓
BMI	0.19 ± 0.02^a^	0.15 ± 0.02^b^	0.16 ± 0.01^cb^	0.16 ± 0.02^db^	0.18 ± 0.02^ea^	0.15 ± 0.003^fb^	4.59^**^	16.84↓	15.21↓	17.36↓	3.15↓	16.84↓
Visceral fat (g)	9.28 ± 0.54^a^	6.01 ± 1.19^b^	2.83 ± 0.46^c^	6.61 ± 1.92^db^	6.33 ± 0.32^eb^	5.62 ± 0.50^fb^	21.35^***^	35.23↓	69.50↓	28.77↓	31.78↓	39.43↓
Visceral fat (%)	5.59 ± 0.37^a^	3.25 ± 0.79^b^	1.29 ± 0.29^c^	3.02 ± 0.59^db^	2.82 ± 0.32^eb^	2.67 ± 0.31^fb^	41.73^***^	41.86↓	76.92↓	45.97↓	49.55↓	52.23↓
Heart weight (g)	0.38 ± 0.05^ae^	0.29 ± 0.05^b^	0.41 ± 0.04^ca^	0.38 ± 0.03^de^	0.34 ± 0.04^ef^	0.33 ± 0.02^fb^	6.32^**^	24.47↓	6.77↑	1.04↓	9.37↓	14.06↓
Heart weight (%)	0.23 ± 0.03^a^	0.153 ± 0.02^b^	0.19 ± 0.006^c^	0.17 ± 0.01^dc^	0.15 ± 0.02^ebd^	0.15 ± 0.008^fbd^	14.00^***^	33.76↓	19.91↓	23.37↓	33.33↓	32.46↓
Liver weight (g)	4.12 ± 0.72=	2.74 ± 0.53^b^	3.44 ± 0.24^c^	2.90 ± 0.29^dbc^	3.22 ± 0.45^ebc^	3.08 ± 0.34^fbc^	5.70^**^	33.49↓	16.50↓	29.61↓	21.84↓	25.24↓
Liver weight (%)	2.45 ± 0.44^a^	1.45 ± 0.16^b^	1.54 ± 0.14^cb^	1.34 ± 0.08^db^	1.42 ± 0.16^eb^	1.45 ± 0.16^fb^	16.95^***^	40.81↓	37.14↓	45.30↓	42.04↓	40.80↓
Kidney weight (g)	0.85 ± 0.17^a^	0.57 ± 0.06^b^	0.69 ± 0.16^cb^	0.61 ± 0.09^db^	0.68 ± 0.08^eb^	0.652 ± 0.03^fb^	3.50^*^	32.86↓	18.31↓	27.69↓	20.18↓	23.47↓
Kidney weight (%)	0.51 ± 0.12^a^	0.30 ± 0.02^b^	0.30 ± 0.04^cb^	0.28 ± 0.03^db^	0.30 ± 0.03^eb^	0.31 ± 0.03^fb^	12.00^***^	40.85↓	40.07↓	44.74↓	41.43↓	39.88↓
Spleen weight (g)	0.16 ± 0.03^a^	0.16 ± 0.03^ba^	0.27 ± 0.04^c^	0.18 ± 0.03^da^	0.21 ± 0.02^ed^	0.21 ± 0.02^fd^	9.16^***^	1.234↑	67.9↑	12.34↑	29.62↑	30.86↑
Spleen weight (%)	0.09 ± 0.02^a^	0.08 ± 0.01^ba^	0.11 ± 0.02^c^	0.08 ± 0.01^da^	0.09 ± 0.01^ea^	0.09 ± 0.008^fac^	3.43^*^	14.58↓	22.91↑	12.5↓	2.08↓	2.08↑

G0 (day 1 initial values); G1 = CO (oconut Oil); G2 = FO (flaxseed Oil); G3 = OO (olive Oil); G4 = SO (sunflower Oil); G5 = BO (Blended Oil); Means/% ± SD; BW 1, body weight at day 1; BW2, body weight at day 42; BMI, Body mass index; TM Index, Total mass index; FER, food efficacy ratio = (Wt. gain/day ÷ Food intake/day) × 100; * D, days; Effect = [(D42-D1)÷D1] × 100; ↑- Percentage Increase; ↓- Percentage Decrease; Means sharing the same letters in a column are not significantly different from each other at *p* < 0.05; *** = Very highly significant at *p* < 0.001; ** = highly significant at *p* < 0.01; * = significant at *p* < 0.05; ns, non-significant at *p* > 0.05.

The results for food intake revealed significant variations among groups for their initial and final food intake values. Final food intake increased a maximum of 20.09 times for the oil blend (30.06 ± 2.07) and a minimum of 9.42 times for the coconut oil group (27.39 ± 4.05) as compared to the day 1 reading. FER was maximum for the sunflower oil group (9.93 ± 1.45) followed by the blend (8.77 ± 1.11), the olive (8.69 ± 1.83), the flaxseed (8.64 ± 1.83), and the coconut (7.31 ± 3.33) groups. Accumulated food intake per trial was significant in differences among all groups with the lowest intake for the coconut oil group (1,150 ± 169.89) and the maximum for the blend group (1262.7 ± 87.12). Similarly, the percentage fat intake was lowest for the sunflower oil group (15.00 ± 1.47), which increased gradually and non-significantly in the flaxseed oil group (15.94 ± 1.10), followed by olive oil (16.64 ± 1.89) and blend (17.43 ± 1.34), and was maximum for the coconut oil group (17.79 ± 3.78).

Naso-anal length made a maximum increase of 16.1 times for the sunflower group (20.20 ± 0.75) and a minimum of 10.92 times for the coconut oil group (19.30 ± 0.27), and this increase was highly significant among groups. On the other hand, the full length was maximum for the flaxseed oil group (37.10 ± 2.43), followed by the olive (37.00 ± 0.35) and the blend (36.40 ± 0.55) groups. The differences in full length were also highly significant among groups. Results for all obesity biomarkers such as the Lee index, Rohrer index, TM index, BMI, and body fat content were all significant among groups, with the Lee index value lower for the blend group (289.60 ± 8.27) and highest for the flaxseed group (297.67 ± 7.03); Rohrer index, TM index, BMI and body fat contents were lowest for the coconut and blend groups. Percentage visceral fat deposits were least in the flaxseed group rats (1.29 ± 0.29), followed by the blend group (2.67 ± 0.31), while these were the maximum for the olive oil group (3.02 ± 0.59). The differences among organ weights in gram and percentage body weights were highly significant. The maximum weight gain for liver weight was recorded for the flaxseed group, whereas the maximum gain in the spleen weight was recorded in the blend group.

### 3.2 Biochemical indicators

The results for all biochemical indicators are presented in Table 4. The results of blood glucose level indicated a noticeable and highly significant decline in blood glucose levels in each oil group when compared to the blood glucose level at day 1 reading after acclimatization. The coconut group revealed a maximum decline in blood glucose levels by 61.85%, followed by blend 56.23%, flaxseed 48.95%, sunflower 43.94%, and olive 29.82% groups. The results for lipid profiling indicated that total cholesterol showed a very significant decrease in oil groups when compared to the day 1 reading. Here, the decline in total cholesterol was maximum for the blended oil group by 57.37%, followed by the coconut, the flaxseed, the olive, and finally the sunflower oil groups. The results for HDL, LDL, and VLDL have been further tabulated in the form of their percentage content in total cholesterol so that the comparison of various oils can be done easily. Results for HDL indicated that the percentage HDL content in total cholesterol was 12.29% at day 1, which increased to 23.07% of total cholesterol in the coconut oil group, 28.76% in the flaxseed group, 29.63% in the olive oil group, 27.78% in the sunflower oil group, and 34.62% in the blended oil group. These revealed maximum levels of HDL for the blend, followed by the olive, flaxseed, sunflower, and finally coconut oil groups. Moreover, this overall increase in HDL cholesterol was a highly significant increase in HDL levels when compared to the day 1 reading of HDL levels in rats. Similarly, LDL levels have shown a very significant decline when compared to day 1 reading. The percentage content of LDL was 42.62% at day 1 reading that reduced to 30.76% in blend, 30.77% in coconut, 43.21% in olive, 43.84% in flaxseed, and 50% in sunflower oil groups. Two oil groups, i.e., coconut and blend, have shown significant reductions in LDL levels when compared to day 1 reading, whereas the flaxseed and olive groups have shown slight increases and the sunflower oil group revealed significantly increased LDL concentrations at the end of the trial. The results for VLDL showed highly significant reductions in all oil groups. The percentage content of VLDL on day 1 was 45.08%, which made a slight increase in the coconut oil group to 46.15% and was significantly decreased in all other groups. The results of TAGs were highest on day 1, which made highly significant reductions in all oil groups. The maximum decline of 68.57% was recorded in the blend group, followed by a 65.39% decline in the flaxseed, 64.13% in the olive, 62.85% in the sunflower, and the least decline of 45.39% in the coconut oil groups. Overall, the total lipid content was maximum at day 1, which was reduced significantly in oil groups. Here, also the maximum reduction in total lipids was seen in the blend group, followed by the flaxseed, the olive, the sunflower, and the coconut oil groups. The comparison among non-HDL cholesterol indicated the peak levels at day 1, which reduced significantly in oil groups with a maximum reduction of 60.92% in the blend, followed by 42.52% in the coconut, 40.22% in the flaxseed, 34.48% in the olive, and 25.29% in the sunflower oil groups.

**Table 4:** Effect of study oils and their blended oil consumption on biochemical biomarkers in high-fat/high-cholesterol diet rats.

	G0	G1	G2	G3	G4	G5	F value	G1 CO%↑	G2 FO%↑	G3 OO%↑	G4 SO%↑	G5 BO%↑
Glucose	200.70 ± 56.61^a^	76.30 ± 13.04^b^	102.10 ± 28.56^cb^	140.36 ± 25.44^dc^	112.12 ± 66.0^ebc^	87.55 ± 24.13^fb^	6.30^***^	61.85↓	48.95↓	29.82↓	43.94↓	56.23↓
TC (mg/dL)	122.00 ± 7.17^a^	65.00 ± 6.44^bc^	73.00 ± 8.74^cd^	81.00 ± 7.04^de^	90.00 ± 8.1^5e^	52.00 ± 9.03^f^	26.07^***^	46.72↓	40.16↓	33.60↓	26.23↓	57.37↓
HDL-C (mg/dL)	15.00 ± 3.31^abf^	15.00 ± 3.39^bf^	21.00 ± 4.47^cdef^	24.00 ± 5.47^de^	25.00 ± 5.47^e^	18.00 ± 2.73^f^	5.20^**^	23.07% 87.71↑	28.76% 134.01↑	29.63% 141.09↑	27.78% 126.04↑	34.62% 181.69↑
LDL-C (mg/dL)	52.00 ± 5.15^a^	20.00 ± 2.55^bf^	32.00 ± 2.45^c^	35.00 ± 2.55^c^	45.00 ± 2.828e	16.00 ± 4.47^f^	45.22^***^	30.77% 27.80↓	43.84% 2.86↑	43.21% 1.38↑	50% 17.32↑	30.76% 27.83↓
VLDL-C (mg/dL)	55.00 ± 6.32^a^	30.00 ± 4.12^b^	20.00 ± 3.08^cde^	22.00 ± 2.92^de^	20.00 ± 3.39^ef^	18.00 ± 2.26^fcd^	65.27^***^	46.15% 2.37↑	27.39% 39.24↓	27.16% 39.75↓	22.22% 50.71↓	34.62% 23.20↓
TAGs (mg/dL)	315.00 ± 34.16^a^	172.00 ± 15.28^b^	109.00 ± 12.23^cdef^	113.00 ± 17.51^def^	117.00 ± 15.24^ef^	99.00 ± 9.19^f^	94.85^***^	45.39↓	65.39↓	64.13↓	62.85↓	68.57↓
Total lipids (mg/dL)	519.00 ± 46.79^a^	302.00 ± 23.24^bde^	255.00 ± 27.175^c^	275.00 ± 27.73^dce^	297.00 ± 30.14e	203.00 ± 26.41^f^	61.19^***^	41.81↓	50.87↓	47.01↓	42.77↓	60.88↓
Non-HDL-C (mg/dL)	87.00 ± 4.26^a^	50.00 ± 3.94^bc^	52.00 ± 4.30^cd^	57.00 ± 1.58^d^	65.00 ± 3.94^e^	34.00 ± 6.40^f^	82.75^***^	42.52↓	40.22↓	34.48↓	25.29↓	60.92↓
AI	2.25 ± 0.76^ae^	1.36 ± 0.20^bf^	1.56 ± 0.28^cb^	1.52 ± 0.35^db^	1.87 ± 0.45^eb^	0.88 ± 0.12^f^	6.25^***^	39.37↓	30.40↓	32.67↓	16.91↓	61.16↓
CoRI	6.99 ± 1.11^a^	4.46 ± 0.71^be^	3.53 ± 0.37^cdef^	3.46 ± 0.47^d^	3.69 ± 0.51^ed^	2.88 ± 0.12^fd^	27.15^***^	36.21↓	49.39↓	50.46↓	47.26↓	58.72↓
CaRI	21.47 ± 3.15^a^	12.03 ± 3.22^b^	5.32 ± 0.91^c^	4.84 ± 1.05^c^	4.76 ± 0.53^c^	5.57 ± 0.70^c^	58.70^***^	43.96↓	75.20↓	77.48↓	77.82↓	74.07↓
AST (U/L)	160.60 ± 47.16^a^	100.40 ± 22.55^b^	142.40 ± 25.30^ca^	95.40 ± 23.66^db^	135.40 ± 31.91^eab^	107.0 ± 36.87^fbc^	3.29^*^	37.48↓	11.33↓	40.59↓	15.69↓	33.37↓
ALT (U/L)	85.20 ± 4.97^a^	38.00 ± 7.31^b^	30.20 ± 4.38	42.20 ± 11.43^db^	36.40 ± 11.36^eb^	36.40 ± 9.09^fb^	29.65^***^	55.39↓	64.55↓	50.46↓	57.27↓	57.27↓
Total protein (g/dL)	1.95 ± 0.30^a^	2.94 ± 0.21^bef^	2.53 ± 0.69^cbdef^	2.33 ± 0.30^daef^	2.70 ± 0.28^ef^	2.61 ± 0.32^f^	3.95^**^	50.76↑	30.15↑	19.58↑	38.76↑	33.94↑
Creatinine (mg/dL)	0.89 ± 0.19^a^	0.55 ± 0.29^a^	0.47 ± 0.42^a^	0.47 ± 0.44^a^	0.54 ± 0.33^a^	0.34 ± 0.16^a^	1.63^NS^	38.34↓	46.86↓	47.08↓	39.24↓	61.66↓
Albumin (g/dL)	2.44 ± 0.43^ad^	1.50 ± 0.59^b^	1.08 ± 0.23^cb^	2.22 ± 0.41^d^	2.61 ± 0.25^d^	2.12 ± 0.52^d^	9.81^***^	38.39↓	57.59↓	9.23↓	6.69↑	13.15↓
BUN (mmol/L)	5.22 ± 0.59^a^	3.42 ± 0.47^bc^	3.10 ± 0.45^bc^	2.44 ± 0.49^c^	3.50 ± 1.05^b^	3.60 ± 1.25^b^	6.94^***^	34.48↓	40.61↓	53.25↓	32.95↓	31.03↓
RBC (10^6^/μL)	5.62 ± 0.31^abc^	5.92 ± 0.19^bde^	5.29 ± 0.25^c^	6.01 ± 0.31^df^	5.72 ± 0.36^ead^	6.32 ± 0.29^f^	7.25^***^	5.33↑	5.87↓	7.01↑	1.88↑	12.45↑
MCV (fl)	53.60 ± 0.51^a^	55.42 ± 1.28^a^	57.64 ± 2.18^b^	55.20 ± 2.20^a^	54.32 ± 0.36^a^	54.00 ± 2.15^a^	3.91^***^	3.39↑	7.54↑	2.98↑	1.34↑	0.74↑
RDW (%)	13.90 ± 1.15^a^	14.44 ± 0.36^bae^	15.84 ± 0.82^c^	14.62 ± 0.38^dae^	14.92 ± 0.53^e^	14.22 ± 0.41^fae^	5.01^**^	3.88↑	13.95↑	5.18↑	7.33↑	2.30↑
HCT (%)	29.90 ± 1.62^a^	32.84 ± 0.55^be^	30.20 ± 3.66^ca^	33.10 ± 2.25^db^	31.12 ± 1.62^eda^	34.10 ± 0.91^fb^	3.54^**^	9.83↑	1.00↑	10.70↑	4.08↑	14.04↑
Platelets (10^3^/μL)	579.60 ± 50.69^a^	629.80 ± 55.29^ba^	620.40 ± 17.81^ba^	662.20 ± 22.33^bc^	596.40 ± 78.03^a^	656.2 ± 21.61^bd^	2.43^NS^	8.66↑	7.04↑	14.25↑	2.89↑	13.21↑
MPV (fL)	8.02 ± 0.43^a^	7.30 ± 0.22^b^	7.50 ± 0.37^cb^	7.62 ± 0.29^db^	7.74 ± 0.22^acd^	7.74 ± 0.18^acd^	3.29^*^	8.97↓	6.48↓	4.98↓	3.49↓	3.49↓
WBC (10^3^/mm^3^)	32.44 ± 2.06^a^	29.60 ± 0.90^bcdf^	29.23 ± 1.49^cdf^	28.74 ± 1.58^df^	23.50 ± 0.60^e^	29.96 ± 1.81^f^	19.31***	8.75↓	0.89↓	11.41↓	27.55↓	7.64↓
Hb.(g/dL)	12.24 ± 0.54^a^	12.22 ± 0.14^ba^	11.22 ± 0.25^c^	12.20 ± 0.20^da^	11.70 ± 0.58^ec^	12.64 ± 0.36^fa^	8.40^***^	0.16↓	8.33↓	0.32↓	4.41↓	3.26↓
MCH (pg)	21.82 ± 0.33^a^	20.60 ± 0.55^bc^	21.24 ± 0.49^ca^	20.32 ± 0.81^db^	20.34 ± 0.23^eb^	20.02 ± 0.66^fb^	7.66^***^	5.59↓	2.65↓	6.87↓	6.78↓	8.25↓
MCHC (g/dL)	40.72 ± 0.26^a^	37.22 ± 0.43^bc^	36.94 ± 0.90^c^	33.52 ± 4.27^d^	37.50 ± 0.36^ec^	37.06 ± 0.42^fc^	7.99^***^	8.59↓	9.28↓	17.68↓	7.91↓	8.98↓
Lymphocytes (10^9^/L)	24.34 ± 1.96^a^	22.40 ± 2.29^ba^	20.24 ± 2.65^cbd^	22.90 ± 2.11^da^	23.40 ± 1.77^ea^	25.18 ± 3.05^fa^	2.67^*^	7.97↓	16.85↓	5.91↓	3.86↓	3.45↑
Granulocytes (10^9^/L)	0.40 ± 0.08^a^	0.20 ± 0.07^bdef^	0.71 ± 0.25^c^	0.23 ± 0.11^da^	0.30 ± 0.15^ea^	0.33 ± 0.11^fa^	8.61^***^	50↓	79.5↑	42↓	25↓	17↓
Monocytes (%)	1.46 ± 0.59^a^	1.02 ± 0.40^ba^	2.16 ± 0.52^c^	1.02 ± 0.43^da^	1.48 ± 0.33^ea^	0.82 ± 0.21^fb^	6.27^***^	30.14↓	47.95↑	30.14↓	1.36↑	43.84↓

G0 (day 1 initial values); G1 = CO (coconut oil); G2 = FO (flaxseed oil); G3 = OO (olive oil); G4 = SO (sunflower oil); G5 = BO (blended oil); TC, total cholesterol; HDL-C, high-density lipoprotein cholesterol; LDL-C, low-density lipoprotein cholesterol; VLDL-C, very-low-density lipoprotein cholesterol; TAG, triacylglycerides; AI, Atherogenic index; CoRI, Coronary risk index; CaRI, Cardiovascular risk index; AST, Aspartate transaminase; ALT, Alanine transaminase; BUN, Blood urea nitrogen; RBC, Red blood cells; Hb, Hemoglobin; HCT, hematocrit; TLC, total leukocytes; MCV, mean corpuscular volume; MCH, mean corpuscular hemoglobin; MCHC, mean corpuscular hemoglobin count; means/% ± SD; * D, days; effect = [(D42-D1)÷D1] × 100; **↑**- Percentage Increase; ↓- Percentage Decrease; Means sharing the same letters in a column are not significantly different from each other at *p* < 0.05; *** = Very highly significant at *p* < 0.001; ** = highly significant at *p* < 0.01; * = significant at *p* < 0.05; NS, non-significant at *p* > 0.05.

Among LFTs done, the results for AST revealed the maximum reading at day 1 which was reduced in oil groups at the end of the trial. Here, the results of AST for the flaxseed and sunflower groups were non-significant when compared to day 1 reading. The maximum reduction was noted in the olive group followed by the coconut and blend groups. Similarly, the results for ALT were maximum on day 1, which reduced significantly in oil groups. The reduction in ALT was maximum for the flaxseed group, followed by the blend, sunflower, coconut, and olive oil groups. The results of RFTs revealed a significant increase in total protein levels and a non-significant decrease in creatinine levels of all oil groups. The results for albumin indicated a significant decrease in the coconut and flaxseed groups, a non-significant decrease in the olive and blend groups, whereas non-significant increase in the sunflower group. The results for blood urea nitrogen indicated a highly significant decline in all oil groups when compared to the day 1 reading.

The results of the hematological analysis revealed highly significant variations in RBCs, MCV, RDW%, HCT, WBS, Hb, MCH, and MCHC. Here, the results for RBCs were increased in all oil groups except for the flaxseed oil group. MCV, RDW%, and HCT increased significantly, whereas platelets made a significant improvement in the olive oil and blend groups, while this increase was non-significant in all other groups. Overall, the results for blended oil were highly significant when compared to day 1 readings for RBCs, HCT, and WBCs. Overall, there was a reducing trend in the readings for WBC, MCHC, and lymphocytes in oil groups.

### 3.3 Cardiovascular risk indices

The results of AI, CoRI, and CaRI have been tabulated in Table 4. The AI was maximum at day 1, which made a highly significant reduction in oil groups. The maximum reduction of 61.16% was recorded in the blend group followed by 39.37% in the coconut, 32.67% in the olive, 30.40% in the flaxseed, and 16.91% in the sunflower oil groups. Similarly, CoRI reduced significantly from day 1 to day 42 in all oil groups. The maximum reduction was 58.72% in the blend, followed by 50.46% in the olive, 49.39% in the flaxseed, 47.26% in the sunflower, and 36.21% in the coconut oil groups. The CaRI was also maximum at day 1, which made highly significant reductions at the end of the trial in the oil groups. Here, the maximum reduction of 77.82% was noted in the sunflower oil group followed by 77.48% in the olive, 75.20% in the flaxseed, 74.07% in the blend, and 43.96% in the coconut oil groups.

### 3.4 Results of echocardiography analysis

**Figure 1:**
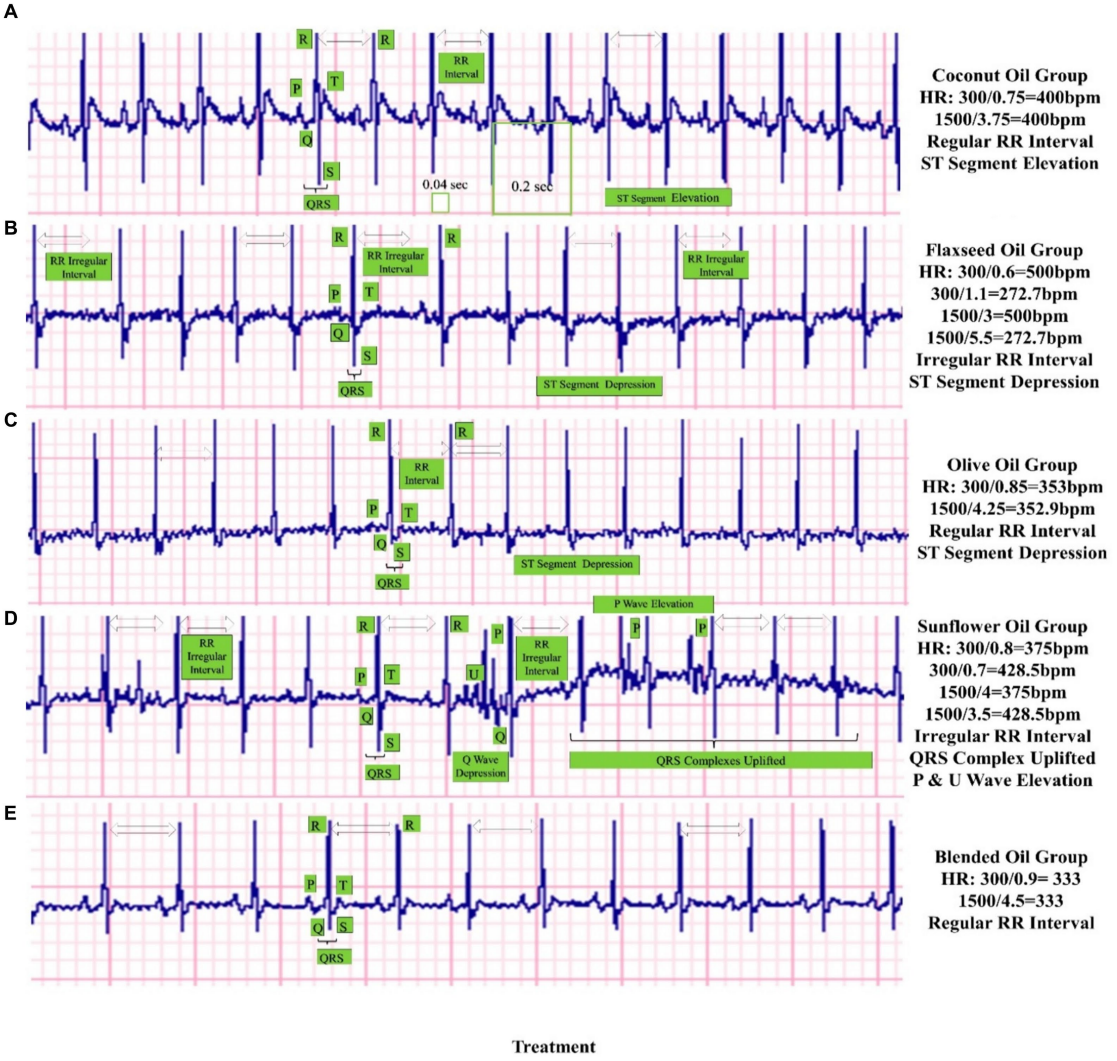
Effect of study oils and blended oil on ECG of high-fat−/high-cholesterol-fed rats. The figure presents the echocardiographs of rats from five groups who were given selected vegetable oils: **(A)** coconut oil, **(B)** flaxseed oil, **(C)** olive oil, **(D)** sunflower oil, and **(D)** their blend as a whole day/100% oil replacement in the diet of rats. The HR (heart rate) has been calculated in bpm, whereas other findings have been indicated on individual graphs such as RR intervals, QRS complex interval, QRS complex uplifting, St segment elevation/depression, and P/U wave elevations. Here, the most profound, smooth, and balanced cardiac rhythms can be noticed in the blended oil ECG.

The results of ECG are shown in Figure 1, indicating that all the study groups almost had a slight alteration in their normal ECG waveforms when considering the placements of their P waves, QRS complexes, RR intervals, and T waves. Overall, a slight abnormal sinus rhythm with ST segments elevation or depression has been observed in rats after feeding the HFHC diet, yet these abnormalities are less prominent in the rats of the blended oil group. There were slight alterations in RR intervals for flaxseed oil and sunflower oil consumption, whereas the QRS complex was prominently uplifted from the baseline in the sunflower oil group along with P and U wave elevations. The slight elevation of the ST segment can be observed in the right precordial leads in healthy individuals, but a prominent elevation can be indicative of underlying cardiac issues (ST-Segment Analysis).^1^ The HR computed from RR intervals indicated a smooth HR of 400 bpm for coconut oil, disturbed HR of 272.7–500 bpm for flaxseed oil, smooth HR of 375 bpm for olive oil, disturbed HR of 375 to 428.5 bpm for sunflower oil, while smooth and quite normal HR of 333 bpm for blended oil. QRS complex duration in seconds starting from the Q wave and ending at the S wave was noted from the graph and converted into milliseconds (ms). QRS interval was computed as 30–40 ms for coconut oil and sunflower oil, 20–30 ms for flaxseed oil and olive oil, and 30–35 ms for blended oil.

## 4 Discussion

This 42-day bio-efficacy trial was carried out on hyperlipidemic, fatty liver, and obese SD rats. Overall, the effectiveness of blended oil in this treatment trial has shown significant health protective ameliorations in all physical, clinical, and cardiac biomarkers, when compared to the outcomes of single vegetable oils. Therefore, it can be concluded that blended edible oil with such FA combinations can bring down the progression of cardiac and metabolic diseases.

Here, the results for body weight gains per trial and per day were highly significant in differences among groups and falls within the normal body weight gain range of 2–2.5 g for SD rats as mentioned (39). On the other hand, the percentage increase in body weight was maximum for the blend group by 111.46%, followed by the sunflower, olive, flaxseed, and coconut oil groups. This significant and maximum yet within normal average body weight gain in clinically stressed rats by the HFHC diet (40, 41) is a supportive sign for the use of blend in diseased conditions. While results for improved FER are again a health protective outcome of oil blend consumption for healthy food utilization in diseased rats. Similarly, the FER ratio has shown no significant differences among various oil groups in studies (42). Among obesity indicators investigated, major indicators including Lee, Rohrer, and TM indices along with body fat content were significantly low for the blended oil group. Moreover, the values for the Lee index are in accordance with the previous studies (43, 44). The results of obesity indices have strengthened the hypothesis that blended oil with cardioprotective nutritional indices and a balanced FA profile can help prevent obesity in hyperlipidemic rats. Although the body weight gain was noted maximum in the blended oil group, this gain was in proportion to the naso-anal length of rats, and hence, this did not increase the obesity indices. The Lee index and BMI made maximum declines of 6.82 and 16.84%, respectively, in the blended oil group. The percentage decrease of 16.9% in the Rõhrer index, 14.75% in the TM index, and 53.12% in body fat content were the second-highest reductions after the coconut oil group. Other results of organ weights have also shown normal growth patterns with medium values for the blended oil group for heart and liver weights. The increased weight gain for the kidney and the spleen in the blended oil group could also justify the improved immunity in these rats. Overall similar significant health outcomes have been reported in previous studies for rats who were fed an oil blend and they exhibited considerable reductions in body weight, fat weight, liver weight, fat ratio, food intake, and energy intake (5).

Similarly, the results of biochemical investigations in terms of laboratory tests also revealed significantly improved biomarkers. The blood glucose level was lowered in the blended oil group when compared to other oil groups, in addition to the coconut oil group rats. Here, it can be assumed that the addition of coconut oil in the blended oil has made this significant decline. Previous research has also suggested that taking coconut oil May help with glycemic management, possibly through the mediation of anti-inflammatory effects of phenolic components. It has been discovered that coconut oil contains phenolic antioxidants, such as caffeic acid, ferulic acid, syringic acid, catechin, and epigallocatechin. Research has indicated that phenolic compounds have anti-inflammatory, immunomodulatory, insulin-sensitizing, and antidiabetic effects (45, 46). Similarly, Vogel et al. (47) supported the idea that consuming coconut oil could lower blood glucose levels. Similarly, the flaxseed oil May also be a possible cause as it has also been linked with dramatically lowering blood glucose levels in previous studies (48). The results for lipid profiles including TC, LDL, and TAGs have also indicated maximum and highly significant decreases, while HDL revealed a maximum increase for blended oil group rats. Here, the overall results of this study for lipid profile are within the normal ranges (49). Moreover, the significantly raised HDL along with considerably lowered *Ω*-6/Ω-3 levels in plasma, liver, and adipose tissues as well as serum triglycerides and LDL cholesterol on the consumption of blend was also justified in a previous study (5). Similarly, another study goes in the favor of blended oil consumption due to its hypolipidaemic effect of significant increases in HDL and significant decreases in TC, LDL, and TAGs in comparison with rats fed single oil (50). The significant ameliorations in all hepatic and renal biomarkers are again a positive indicator of the anti-inflammatory and hepatoprotective potential of these individual oils. The blended oil, in particular, has exhibited more protective and balanced outcomes, which May be the result of the combined action of the natural bioactive ingredients included in these different oils, such as the flaxseed, coconut, and olive oils (51–53).

Among cardiovascular risk indices, again the blended oil revealed maximum reductions for AI and CoRI. These maximum reductions are a true indicator of its cardiac protection property, which is probably due to the balanced FA proportions in it in terms of saturated, monounsaturated, polyunsaturated, and Ω-3 FA. The values of AI and CoRI are close to those of Salah et al. (27) and goes in correspondence with the previous results of Famurewa et al., (35) by showing a decline in AI and CoRI of blended oil groups as compared to single oil groups. On the other hand, the outcomes for CaRI were not supportive of the blend, which might be due to the addition of saturated fats from coconut oil. However, as the majority of the saturated fats in this blend are MCFA mainly lauric acid (C12 = 48%) from coconut oil, which is readily absorbed by the liver and oxidized for energy, prevents obesity, reduces hepatic stress, and significantly elevates serum HDL-C as compared to vegetable oils (1). Therefore, this CaRI outcome is still debatable as the saturated fats in the blend are mainly MUFA from coconut oil. Here, in this study the result of ECG further goes in support for the regular consumption of this blended oil. Rat ECG is a commonly used experimental technique in fundamental cardiovascular research (36). Normal HR of SD rats falls 239–508 bpm (36), and the HR of all groups in this study falls in this range and is also in accordance with the previous study (37). QRS intervals of SD rats were reported to be 12–22 ms (36), and the results for QRS interval in this study were on higher sides from this normal range but are nearly in accordance with the previous study (37). The graphical presentations and the calculations of QRS interval, RR interval, and HR from results for ECG have shown normal sinus and cardiac rhythms, therefore supporting the health protectiveness of blended oil in daily meal consumption. Our scientific limitations could be the failure to perform genetic-level investigations and the effect of blended oil consumption on the FA profile of body fats due to the lack of resources.

## 5 Conclusion

The consumption of fats from saturated, trans, and monotonous origins is the main component of current dietary lifestyles that needs to be modified. Combining various FAs in an innovative way to yield a health-protective FA combination could be used as an easy, economical, and health-promoting approach for food industrialists and health practitioners. This study evaluated the therapeutic effects of an oil blend that was developed to obtain such a health-protective FA combination. A 42-day bio-efficacy trial was carried out on 30 SD rats that were first given an HFHC diet to elevate their normal cholesterol levels and induce fatty liver and obesity. Overall, the effectiveness of blended oil in this treatment trial has shown significant health protective ameliorations not only in the ECG of rats but also in the physical and biochemical parameters studied. In a nutshell, the daily consumption of blended oil made from coconut, flaxseed, olive, and sunflower oil in the given ratio could not only protect from the onset of such metabolic diseases, but also it can help in the reversal of these metabolic imbalances caused by the consumption of fatty or processed foods. In conclusion, the consumption of blended oil to replace regular single oils can be a useful strategy for enhancing overall health outcomes. Future recommendations based on this research May involve developing different oil blends with different fats or oils, looking into the genetic outcomes of various clinical biomarkers, and launching these blends into the market.

## Data Availability

The original contributions presented in the study are included in the article/supplementary material, further inquiries can be directed to the corresponding author.

## Footnotes

^1^ my-ekg.com
